# Application of Internet of Things Combined with Wireless Network Technology in Volleyball Teaching and Training

**DOI:** 10.1155/2022/8840227

**Published:** 2022-08-10

**Authors:** Tao Zhang, Chenying Jiao, Hui Sun, Xiaolong Liang

**Affiliations:** ^1^Department of Physical Education, Dongguan City College, Dongguan 523109, Guangdong, China; ^2^Physical Education Department, Longcheng High School, Shenzhen 518106, Guangdong, China

## Abstract

Motion information collection technology is a means for measuring, tracking, and recording the movement traces of individuals in space. This method can complete the data collection of volleyball players and the ball trajectory and realize quantification and statistical analysis of the data to present a virtual model of the player's movement trajectory. It is inseparable from the acquisition of information to complete the information collection. Therefore, this work uses the radio frequency identification (RFID) technology in the Internet of Things technology to build an information collection system and apply it to volleyball sports. The existing positioning system based on RFID has problems such as significant positioning errors and high system costs due to the arrangement of a large number of readers. This paper first introduces the theoretical knowledge of the RFID system, wireless network positioning technology, and RFID system positioning method in the Internet of Things. Besides, the theoretical framework of the volleyball movement information acquisition system is presented based on the Received Signal Strength Indication of the RFID system and Location Identification based on the Dynamic Active RFID Calibration (LANDMARC) algorithm. Then, the LANDMARC algorithm is improved through the Centroid Positioning algorithm, forming the CP-LANDMARC algorithm. Finally, a simulation experiment is conducted to test the system effect. The results demonstrate that: (1) the average error of the basic LANDMARC algorithm is 0.55 meters, and the average error of the CP-LANDMARC algorithm is 0.46 meters; (2) the average error of the CP-LANDMARC algorithm is 0.43 meters when the reference label is set to be evenly distributed in a square, and the average error of the optimized algorithm is 0.38 m when the reference label is set as an equilateral triangle; and (3) when the number of reference labels increases to 110, the average error decreases from 0.38 to 0.29. This paper aims to improve the quality of volleyball teaching and training by designing a relevant sports information acquisition system.

## 1. Introduction

Motion information collection technology is a means of measuring, tracking, and recording the movement of individuals in space [1]. This method can collect the trajectory data of volleyball players and the ball and, in the meanwhile, realize the quantification and statistical analysis of the data to establish and present the virtual model of the player's movement track [2]. In addition, the real-time coordinate information can assist coaches and players in evaluating tactics to realize the scientific and modernization of volleyball training [3]. With the continuous reform of competition rules, sports such as volleyball have to pay close attention to the optimization of technology and tactics. The accelerating speed of the ball and the tactical transformation have significantly intensified the confrontation between offense and defense. Correspondingly, various strengthened qualities of volleyball players and coaches are essential for victories, such as physical function, ball skills, and coaching ability. Therefore, a clear and direct presentation of the sports information of volleyball players is a vital part of enhancing the ability of volleyball players [4].

At this stage, discussions about wireless communication technology emerge in an endless stream, making significant advances in the technology and extending its application scope [5]. The principal working principle of wireless positioning technology is to measure and receive multiple wireless signals through the instrument and then construct the positioning equation system according to the geometric constraints to determine the coordinate position of the measured object [6]. In the instrument measurement process, the measured variables primarily contain the signal transmission time, such as the difference between the arrival times of different signals, the signal amplitude, the phase arrival angle, and the received signal strength indication [7]. In recent years, radio frequency identification (RFID) technology, ultra-wide band technology, and ZigBee technology have been the research hotspots of scholars. Specifically, RFID is an advanced fusion of wireless communication technology and automatic identification technology [8]. The changes brought by RFID technology will definitely impact everyone's lives and become one of the most influential wireless communication technologies after third generation technology in the near future. At this stage, spatial orientation and position tracking are critical parts of the RFID technology. Both RFID and tracking algorithms use electronic tags to independently identify the characteristics of objects. In other words, the radio frequency signal strength information between the reader and the electronic tags is used to measure the spatial position of the object. Due to the characteristics of low investment, high precision, real-time performance, and considerable transmission distance, this technology has applicability to real-time dynamic information collection in competitive sports [9]. At present, there are many location algorithms based on RFID. These algorithms first embed the electronic tag inside the object or place it on the surface of the object and then place a certain number of readers in known positions. The object containing the electronic tag enters the area of effect of the reader. Finally, the readers automatically record the location information of the electronic tag. In this process, the position of the reader can be regarded as the coordinates of the tag [10]. The Location Identification based on Dynamic Active RFID Calibration (LANDMARC) system is a positioning system based on received signal strength information. The reader in this system can roughly measure the energy level of the tag's transmitted signal reaching the reader, preliminarily calculate the power level to obtain the corresponding distance information, and finally, find the tag position by geometric theory [11]. However, this positioning mode has certain defects. A complex and changeable space environment will significantly reduce its accuracy, resulting in the inability to meet user requirements. Setting some fixed reference labels to assist the positioning task can remove the negative factors brought by the environment and enhance the system stability and accuracy [12].

According to the research literature, the current RFID positioning technology and motion information acquisition systems have been developed to a certain extent. Still, the existing RFID-based positioning algorithm has a large positioning error and a huge expense caused by the allocation of massive readers. This work builds a volleyball sports information acquisition system based on RFID technology and develops the CP-LANDMARC algorithm. The innovation of this work lies in the optimization of the LANDMARC algorithm by using the Centroid Localization algorithm to reduce the errors of the volleyball information acquisition system. This paper aims to improve the sports information acquisition system to assist teachers in completing high-quality volleyball training activities by utilizing and optimizing the existing positioning algorithm.

## 2. Methods

### 2.1. Operating Rationale of RFID Systems

#### 2.1.1. Composition of RFID Systems

The RFID system is primarily composed of two technological parts: electronic tags and readers. Other parts, such as computers, networks, and wireless devices, can be added based on these two components to work together and constitute a complete solution [13]. Electronic tags are tools to store attribute information of objects, which are generally placed on the surface or inside of objects and composed of antennas and electronic chips [14]. Readers are responsible for recording the information contained in the tag and inserting information into the storage unit in the tag. Its equipment is supported by both hardware and software. The hardware includes two modules: the main controller and a radio frequency processor. The function of antennas and other circuit software parts is to respond to the action command sent by the tag and the received command [15]. In addition, readers use the antenna to transmit the data to be transmitted after encoding and processing. They also receive the electronic tag signal within a recognizable distance, then employ the internal circuit to decode it, and finally judge whether to send back a response according to the information [16]. Moreover, it can use an external interface to transfer the acquired data or processed data to other devices. For instance, the reader will complete the corresponding work on the electronic tag after obtaining the instructions from the host computer. The communication between the reader and the electronic tag is carried out by means of coupling, including inductive coupling and electromagnetic backscattering coupling. Using this method can efficiently complete energy transportation and information sharing between the two. In addition, the reader is slightly more complicated than the electronic tag, requiring a higher fund investment [17].

#### 2.1.2. Classification of Electronic Labels

**Figure 1:**
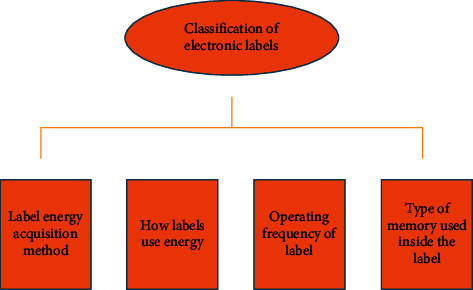
Classification of electronic tags.

Figure 1 displays the categories of electronic tags in different ways.

**Figure 2:**
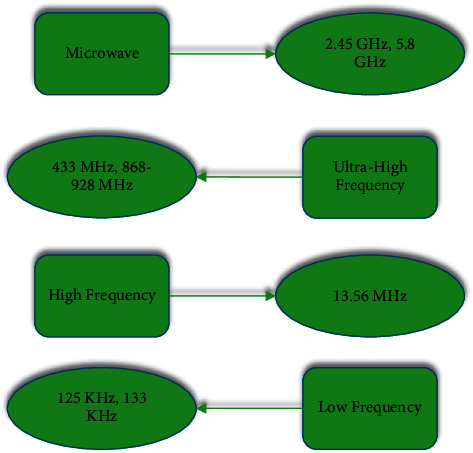
RFID systems in different frequency bands.

1. Electronic tags can be divided into active tags, passive tags, and semiactive tags according to the different energy acquisition methods [18].
2. Electronic tags can be divided into active tags, passive tags, and semiactive tags according to the way they use energy [19].
3. Electronic tags can be classified into low-frequency, high-frequency, ultra-high-frequency, and microwave tags according to their operating frequencies [20]. In addition, electronic tags will exhibit diverse characteristics when performing tasks on various frequency bands.  Figure 2 reveals the specific frequency band distribution of electronic tags.
4. According to the different storage devices contained in the tag, electronic tags can be divided into read-only tags and readable and writable tags [21].

#### 2.1.3. Collision Prevention Technology

**Figure 3:**
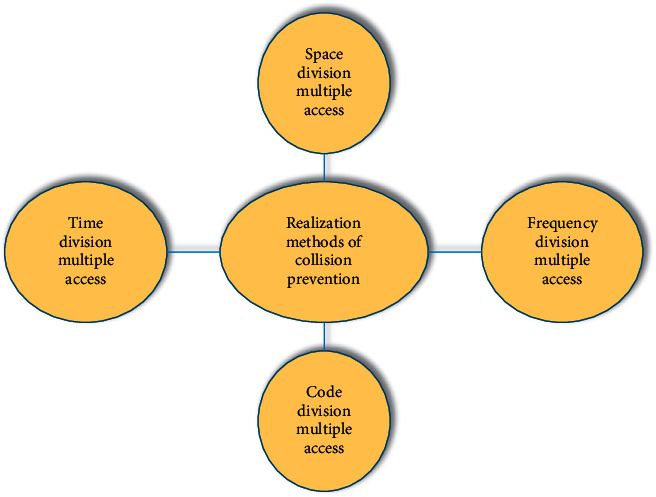
Implementation approaches of the collision prevention technology.

Collision is a common phenomenon in RFID, and the reason is that various devices will interfere with each other during the operation of RFID. The collision can be avoided by collision prevention. There are two different collisions in the RFID system: the collision generated by the reader and the collision generated by the electronic tag [22]. The collision prevention method can eliminate the collision between tags or readers in four ways [23], as shown in Figure 3.

### 2.2. Wireless Network Positioning Technology

#### 2.2.1. Global Positioning System

Global positioning system (GPS) positioning technology is often used in military and civilian fields because of its high positioning accuracy and wide range of functions. The technology consists of differential GPS and auxiliary GPS.

1. Differential GPS: the working principle of differential GPS is to locate itself through the receiving device, make a difference with the measured data to obtain error data based on the known coordinate data, and finally use this data to correct the terminal device location data. In this process, terminal devices in the same area can share the error data. Overall, the accuracy of differential GPS is higher than that of conventional GPS.
2. Auxiliary GPS: the auxiliary GPS' reference network can send the positioning information to the mobile platform through the wireless network during the positioning process. The magnitude of the time is downgraded to seconds in the whole process [24].

#### 2.2.2. Indoor Positioning Technology

**Figure 4:**
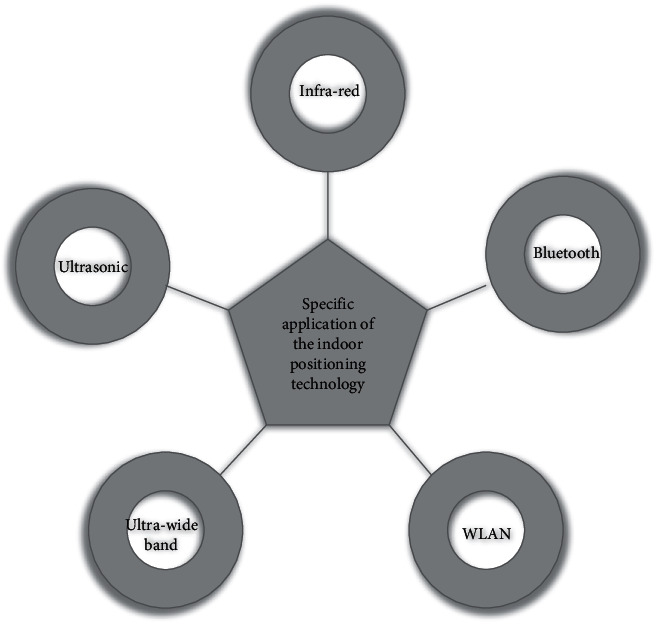
Illustrates the specific applications of the existing indoor positioning technology.

The specific application of the current indoor positioning technology is shown in Figure 4.

### 2.3. RFID Positioning Method

The RFID technology method used here is the received signal strength indication (RSSI) method based on the received signal strength [25]. Electromagnetic waves are in a random propagation state. Here, the distance between the transmitting device and the receiving device is denoted as *R*_*i*_, and the receiving power of the receiving device is marked by *P*_*r*_*i*__. The equation for receiving power of the receiving device is given as follows:

(1) $$\begin{array}{c} P_{r_{i}}=\frac{P_{t}\cdot G_{t}\cdot G_{r_{i}}\cdot \lambda^{2}}{4\pi R_{i}^{2}}. \end{array}$$

In Equation (2), *λ* refers to the wavelength of the electromagnetic wave; *P*_*t*_ represents the signal transmission power of the tag; *G*_*t*_ and *G*_*r*_*i*__ denote the increment of the tag and the antenna's increment of reader *i*, respectively. According to Equation (1), the distance *R*_*i*_ between the tag and the reader can be obtained by using the measured value *P*_*r*_*i*__.

**Figure 5:**
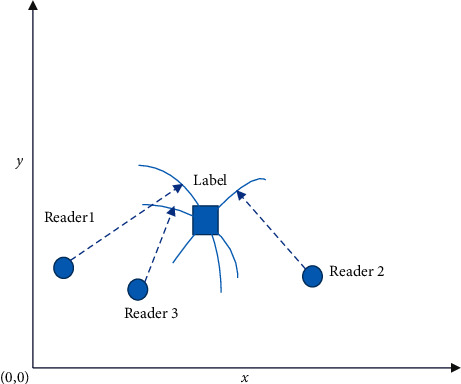
RSSI positioning method.

Figure 5 presents the coordinate system established here.

Figure 5 suggests that three readers can be used for tag positioning operations. The area where the intersection of the three circles with the positions of the three readers as the center converge is the position of the tag, where *R*_1_, *R*_2_, and *R*_3_ are the radii of the three circles [26].

In indoor scenarios, the average transmitted signal power will decrease as the distance increases according to theoretical propagation models and experiences. Therefore, in the positioning system, the trajectory loss empirical model can be defined as follows:.

(2) $$\begin{array}{c} P=P_{0}+10n\;\log_{10}\left(\frac{d}{d_{0}}\right)+X_{\sigma }. \end{array}$$

In Equation (2), *n* represents the path loss index; *d*_0_ signifies the reference distance; *d* stands for the distance between the reader and the electronic tag; *P*_0_ represents the signal size of the electronic tag with the reference distance *d*_0_ received by the reader; *P* signifies the signal size of the electronic tag with the distance *d* received by the reader; *X*_*σ*_ stands for a normal random variable with the mean of 0 and the standard deviation of *σ*.

### 2.4. LANDMARC Positioning Algorithm

As a typical indoor positioning algorithm based on signal strength information, the LANDMARC positioning algorithm has the characteristics of high stability, positioning accuracy, and applicability [27]. The core of the LANDMARC algorithm is to incorporate redundant fixed reference labels into the positioning device to help locate the label. In this process, the signal strength value of the tag to be positioned received by the reader is compared with the signal strength value of the reference tag to solve the coordinates of the tag to be positioned [28].

In addition, the reference tags with the smallest difference in signal strength values cannot be used due to the tiny difference between the signal strength values of the electronic tags obtained by the reader. Therefore, the LANDMARC algorithm uses the received signal strength difference to locate the nearest reference tags to the required tag [29].

If the RFID contains *n* readers, *m* reference tags, and *u* unidentified tags, the specific operation steps of the LANDMARC algorithm are the follows.

(1) The signal magnitude vector of the reference label is set as $\overset{⟶}{\theta }=\left(\theta_{1},\theta_{2},\ldots ,\theta_{n}\right)$; the signal strength vector of the unlocated tag is set as $\overset{⟶}{S}=\left(S_{1},S_{2},\ldots ,S_{n}\right)$. Thus, the Euclidean distance between them can be written as follows:
  (3) $$\begin{array}{c} E_{j}=\sqrt{\overset{n}{\underset{i-1}{\sum }}{\left(\theta_{i}-S_{i}\right)}^{2}}. \end{array}$$
In Equation (3), *θ*_*i*_ and *S*_*i*_ represent the signal size of the reference tag and the unidentified tag obtained by the reader *i*, and *i*=(1,2,…, *n*); *E*_*j*_ refers to the Euclidean distance between the two. The smaller the distance, the smaller the distance between the two. Besides, *j*=(1,2,…, *m*).
(2) The vector is denoted as $\overset{⟶}{E}=\left(E_{1},E_{2},\ldots ,E_{m}\right)$ according to the *E*_*j*_. The *k* reference labels with the smallest Euclidean distance from the set are determined, which are the *k* nearest neighbors of the unlocated label.
(3) The coordinates of the unidentified label is calculated through weighted calculation according to the known coordinates of the *k* nearest positions.

(4) $$\begin{array}{c} \left(x,y\right)=\overset{k}{\underset{l=1}{\sum }}w_{l}\left(x_{l},y_{l}\right). \end{array}$$

In Equation (4), *w*_*l*_ represents the weight (*l*=1,2,3,…, *k* < *m*) of the *l*-th nearest position, which can be expressed as follows:.

(5) $$\begin{array}{c} w_{l}=\frac{\left[1/E_{l}^{2}\right]}{\left[\sum_{l=1}^{k}\left(1/E_{l}^{2}\right)\right]}. \end{array}$$

The positioning algorithm calculates the theoretical coordinate value (*x*, *y*) of the unlocated tag. The error *e* between (*x*, *y*) and the given actual coordinate value (*x*_0_, *y*_0_) can be expressed as follows:.

(6) $$\begin{array}{c} e=\sqrt{{\left(x-x_{0}\right)}^{2}+{\left(y-y_{0}\right)}^{2}}. \end{array}$$

### 2.5. Improvement of the LANDMARC Positioning Algorithm

**Figure 6:**
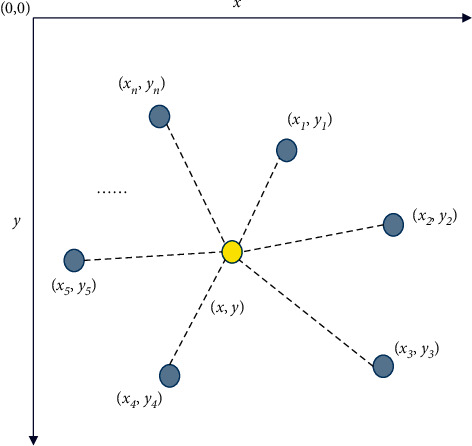
Centroid positioning algorithm.

In practical applications, on the one hand, the wireless signals will be reflected, diffracted, refracted, and absorbed due to the complexity of the application scene and system structure and the high-speed movement of athletes; on the other hand, the signal strength information of the space and the coordinate information of the geometric space are solved by the same expression, which cannot ensure the applicability and accuracy of the algorithm. Therefore, this work uses the centroid positioning algorithm to optimize the LANDMARC algorithm. The working principle of the centroid positioning algorithm is to set the geometric centroid of all beacon nodes connected to the unlocated node as the approximate coordinates of the unidentified node [30].  Figure 6 shows the two-dimensional centroid positioning algorithm.

In the positioning process of the algorithm, the beacon node sends beacon information to neighboring nodes at regular intervals. When the size of the information obtained by the undetected node from each beacon node exceeds a certain value, the node is in a communication state with the beacon node until obtaining all the beacon nodes communicating with it. Then, the coordinate value of the unlocated node can be expressed as follows:

(7) $$\begin{array}{c} \left(x,y\right)=\left(\frac{\sum_{i=1}^{k}x_{i}}{k},\frac{\sum_{i=1}^{k}y_{i}}{k}\right). \end{array}$$

In Equation (7), (*x*_*i*_, *y*_*i*_) represents the position of the beacon node connected to the unidentified node.

If the undetected node contains three beacon nodes within its communication distance, the weighted Centroid Positioning algorithm is used to calculate the coordinate value of the unlocated node according to the following equation:

(8) $$\begin{array}{c} x_{i}=\frac{\left(x_{1}/d_{1}+d_{2}+x_{2}/d_{2}+d_{3}+x_{3}/d_{3}+d_{1}\right)}{\left(1/d_{1}+d_{2}+1/d_{2}+d_{3}+1/d_{3}+d_{1}\right)},\\ y_{i}=\frac{\left(y_{1}/d_{1}+d_{2}+y_{2}/d_{2}+d_{3}+y_{3}/d_{3}+d_{1}\right)}{\left(1/d_{1}+d_{2}+1/d_{2}+d_{3}+1/d_{3}+d_{1}\right)}. \end{array}$$

Where (*x*_1_, *y*_1_), (*x*_2_, *y*_2_), and (*x*_3_, *y*_3_) represent the coordinates of different beacon nodes; *d*_1_, *d*_2_, and *d*_3_ signify the distance between the unidentified node and different beacon nodes. The size of the factors 1/*d*_1_+*d*_2_, 1/*d*_2_+*d*_3_, and 1/*d*_3_+*d*_1_ reflect the influence ability of different beacon nodes to the position of the unlocated node.

Furthermore, the LANDMARC algorithm is optimized through the centroid positioning algorithm. First, the empirical loss model is used to map the relationship between the received signal strength and the geometric path. Second, efficient reference labels are utilized to form the set *R* to detect the positions of the *k* nearest undetected label. The number of constructed triangles is as follows:

(9) $$\begin{array}{c} k^{\prime }=C_{k}^{3}. \end{array}$$

**Figure 7:**
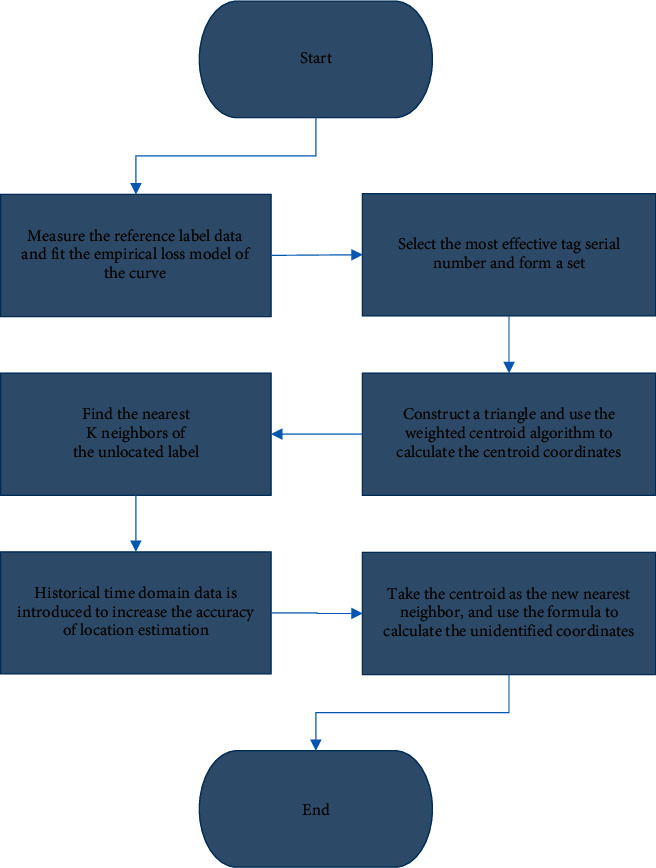
Optimization steps of the LANDMARC algorithm.

Then, the weighted centroid positioning algorithm is used to obtain the information of *k*′ centroid coordinates. Then, the *k*′ centroids replace the nearest position of the new unlocated tag. Besides, the historical time domain data is added to avoid the conflict between different readers and different electronic tags. In addition, the effect of random factors of correlation between data on positioning accuracy is used to obtain better position coordinates.  Figure 7 displays the specific algorithm flow.

### 2.6. Framework Design of the Volleyball Information Acquisition System

**Figure 8:**
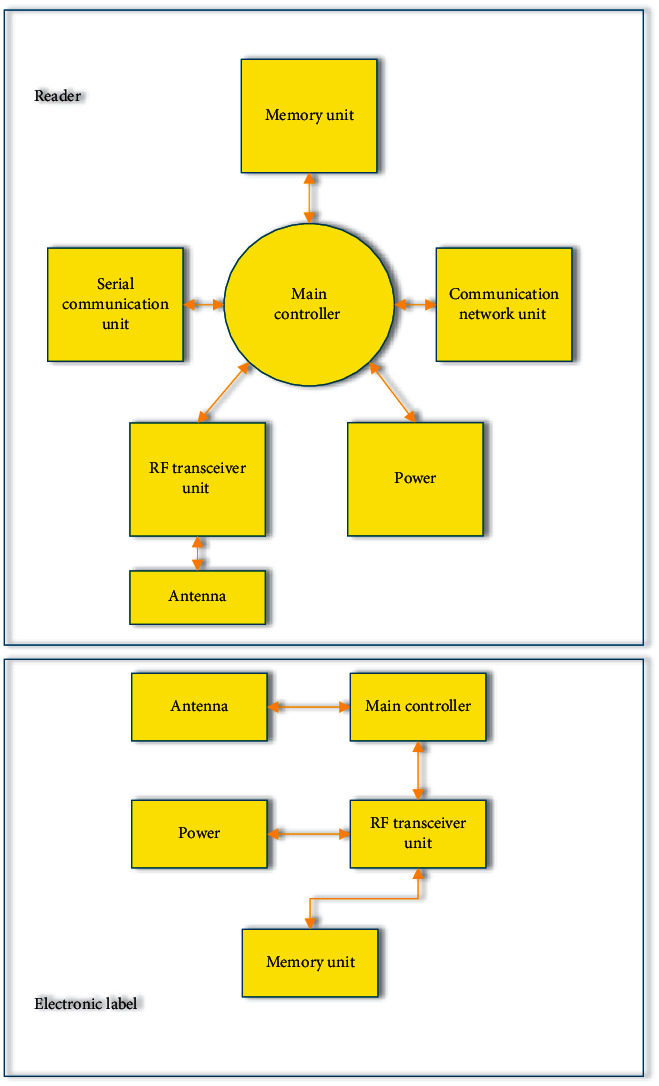
Content architecture of the RFID volleyball information acquisition system.

According to the above theoretical content analysis, the hardware structure of the volleyball sports information acquisition system based on RFID technology contains the reader structure and the electronic label structure.  Figure 8 the content structure of the volleyball sports information acquisition system constructed here.

### 2.7. Simulation Experiment Design

The volleyball court is set to different shapes, and a fixed number of RFID readers and different reference tags are used in the system. An electronic tag is attached to each volleyball player. The experimental data comes from the on-site readings of the reader. The cumulative distribution function is used in the simulation experiment to represent the probability distribution of the positioning error *e*.  Table 1 lists the specific experimental parameters.

## 3. Results and Analysis

### 3.1. The Optimization Effect of the LANDMARC Algorithm

**Figure 9:**
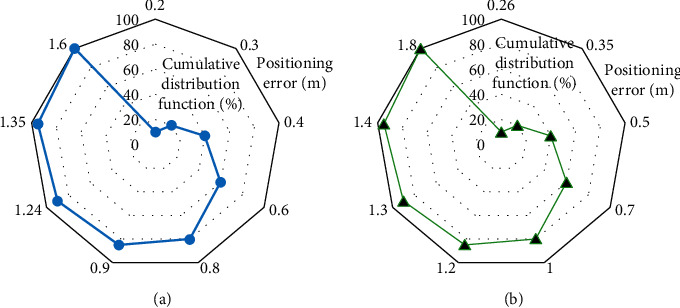
Simulation results of the basic algorithm and improved algorithm. (a) The system positioning performance under the basic LANDMARC algorithm; (b) the system positioning performance under the CP-LANDMARC algorithm.

There are two square volleyball courts with a length of 9 meter, four readers, and 49 reference tags. Besides, four readers are placed on the diagonal of the square. The value of *k* is set to 4.  Figure 9 provides the simulation results of the basic LANDMARC algorithm and the CP-LANDMARC algorithm.

Figure 9 shows that the cumulative distribution function images under the basic LANDMARC algorithm and the improved LANDMARC algorithm are on the rise. According to the calculation, the average errors of the basic algorithm and the improved algorithm are 0.55 m and 0.46 m, respectively. The above data shows that the positioning effect of the improved LANDMARC algorithm is better than that of the basic algorithm.

### 3.2. Localization Performance for Different Distributions of Reference Labels

**Figure 10:**
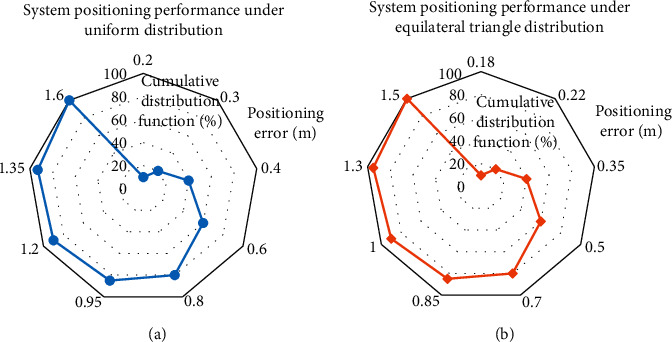
Localization performance for different divisions of reference labels, (a) system performance under uniform distribution; (b) system performance under equilateral triangular distribution.

The volleyball training area is divided into several equilateral triangles with a side length of 1.5 meters. Four RFID readers are placed at the endpoints of different fields, and 56 reference labels are set according to the characteristics of the equilateral triangle. Meanwhile, six unlocated tags are set on the inside of all volleyballs.  Figure 10 provides the results.

Figure 10 suggests that the cumulative distribution function images under different distributions of reference labels all show an upward trend. According to the calculation, the average errors of the uniform distribution of the reference labels in a square and the improved uniform distribution in an equilateral triangle are 0.43 m and 0.38 m, respectively. The above data show that the improved algorithm using the equilateral triangle distribution model is more effective.

### 3.3. Simulation Effects of the Number of Different Reference Positioning Tags

**Figure 11:**
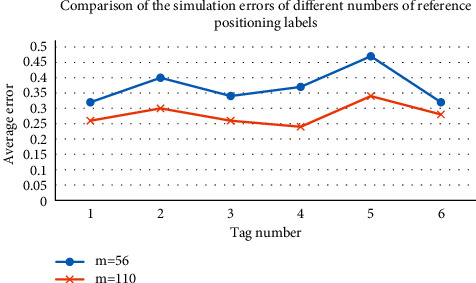
Simulation results for different numbers of reference positioning tags.

When the side length of the equilateral triangle is set to 1 meter, that is, when the distance between the reference positioning labels is shortened, the number of reference positioning labels in the field needs to be added to 110, and the reference labels are about twice as dense as before.  Figure 11 shows the simulation results.

Figure 11 demonstrates that when the number of reference labels increases to 110, the average error decreases from 0.38 to 0.29. This result demonstrates that changing the number density of spatial reference labels can change the positioning accuracy. Within a certain label density, the greater the density, the greater the positioning accuracy. However, when the number density of labels increases to a certain value, the change in accuracy will be insignificant. Therefore, an appropriate value must be selected for the setting of the number of reference labels to reduce errors in practical applications.

## 4. Conclusion

This work compares the advantages and disadvantages of the existing information acquisition systems based on the development status of sports information collection systems. Besides, RFID technology is innovatively applied to the information collection system for volleyball, combined with wireless positioning technology. Moreover, a volleyball sports information acquisition system is designed based on the relevant theoretical support of sports information acquisition systems and RFID positioning technology. This model realizes precise positioning in two-dimensional space and meets the requirements of the volleyball information acquisition system for coordinate information. Furthermore, an improved LANDMARC algorithm is developed based on the previous research on information acquisition systems and by combining the classic algorithm with a wireless sensor algorithm, dramatically improving the positioning accuracy. The distribution of readers and reference tags affects the positioning accuracy. The equilateral triangle distribution model in wireless sensor networks is applied to the improved LANDMARC algorithm, improving the overall positioning accuracy of the system. The system is analyzed by setting up a simulation experiment. When different algorithms and different experimental environments are used to verify the effect of the system, the average error of the system is different, but the overall value is small. The results indicate that the information acquisition system has high precision. Still, this work has some deficiencies. The information acquisition system designed here is only verified through simulation experiments without the actual volleyball training. The subsequent study will apply the system to actual volleyball training to further verify its performance. On the whole, the system proposed here has many advantages, such as a wide range of functions, a large number of acquisitions, low power consumption, anti-interference, and low cost, which will play an important role in promoting sports competitions and training.

## Figures and Tables

**Table 1:** Experimental parameter settings.

Experimental parameter	Experiment 1	Experiment 2	Experiment 3
Volleyball court shape	Square	Equilateral triangle	Equilateral triangle
Ground side length	9 m	1.5 m	1 m
Number of readers	4	4	4
Number of reference labels	49	56	110
Undetermined label	6	6	6

## Data Availability

The data used to support the findings of this study are available from the corresponding author upon request.
